# Owner Personality, Owner-Dog Attachment, and Canine Demographics Influence Treatment Outcomes in Canine Behavioral Medicine Cases

**DOI:** 10.3389/fvets.2020.630931

**Published:** 2021-01-22

**Authors:** Lauren Powell, Darko Stefanovski, Carlo Siracusa, James Serpell

**Affiliations:** School of Veterinary Medicine, University of Pennsylvania, Philadelphia, PA, United States

**Keywords:** dog behavior, human-dog attachment, personality, veterinary behavior, behavior problems

## Abstract

Human and canine parameters can affect the development of canine behavior problems, although their influence on the dog's response to veterinary behavioral treatment remains unclear. This study aimed to investigate the possible associations between canine behavior following clinical intervention and canine demographic characteristics, owner personality and owner-dog attachment. The study included 131 dog-owner dyads who attended a veterinary behavioral service. Owners completed the C-BARQ at baseline, 3-months and 6-months, and the 10 Item Personality Inventory and Lexington Attachment to Pet Scale at baseline. Data were analyzed for the effect of clinical intervention on C-BARQ subscale scores using mixed effect models. Binary logistic regression models were used to analyze the association between behavior change and canine and owner parameters. Within 6-months of veterinary consultation, trainability increased (coefficient 0.03, *p* = 0.01) and chasing (coefficient −0.04, *p* = 0.02), separation-related behavior (coefficient −0.04, *p* = 0.01) and energy level (coefficient −0.04, *p* = 0.05) decreased. Treatment outcomes were associated with both canine and owner variables. Canine behavior at baseline was the most consistent predictor of behavior change with less desirable baseline behavior associated with greater odds of decreased problem behavior at three- and 6-months post-consultation across most C-BARQ subscales. Canine age and weight; owner conscientiousness, extraversion and openness; and owner-dog attachment were also associated with treatment outcomes for some behavioral categories. These findings could be used by veterinarians to formulate more accurate prognoses and provide owners with targeted advice to reduce the influence of background factors on the dog's response to clinical behavioral intervention.

## Introduction

Dogs are the most common companion animal in the United States with a canine population of approximately 77 million (1). Most owners report high levels of satisfaction with (2, 3) and attachment to their dogs (4, 5). Yet, an estimated 3.3 million dogs are relinquished to animal shelters in the US each year, of which 670,000 (22%) are euthanized (6). Although the reasons for relinquishment vary between communities (7, 8), behavior problems are consistently documented as a leading or contributing factor (8, 9). Behavioral disorders also contribute to premature mortality among privately owned dogs due to euthanasia (10, 11), with a study of electronic veterinary records in the UK indicating behavioral problems were the most frequent cause of death in dogs under the age of three (11).

Canine behavior and temperament are influenced by a multitude of owner and dog characteristics, including dog age, sex and weight (12–14), owner personality and owner-dog attachment (15–20). For example, dog age has been inversely associated with behaviors such as trainability and sociability (21). Male dogs have been found to exhibit higher levels of aggression and separation-related behavior compared with female dogs who tend to exhibit greater fearfulness (13, 22). Considering owner characteristics, Podberscek and Serpell (18) found owners of aggressive dogs were more likely to be emotionally unstable, shy, tense and undisciplined. Confirming these findings, Gobbo and Zupan (20) found higher levels of neuroticism among the owners of aggressive dogs (20). Dodman, Brown et al. (19) found higher levels of stranger-directed fear among dogs whose owners scored low in extraversion, conscientiousness and emotional stability (19). Owner-dog attachment has also been associated with canine behavior, with Konok, Kosztolányi et al. (15) reporting increased separation-related behavior among dogs of owners with insecure-avoidant attachment styles. A positive correlation has also been documented between separation-related behavior, trainability and the strength of owner-dog attachment (17).

Despite recognition of the multifactorial nature of canine behavior, it is not clear how owner or dog parameters influence treatment outcomes in canine behavioral medical cases. Treating behavior problems is time-consuming and often requires owners to make considerable lifestyle changes (23), implement behavioral modification techniques (24) and administer psychoactive medications (25–27). Moreover, owner compliance is a significant challenge for veterinarians in clinical behavioral medicine (28). Understanding the influence of background parameters on the canine behavioral response to clinical intervention could help veterinarians predict treatment outcomes and provide targeted advice to owners. It may also help to streamline the process of canine behavioral diagnosis. Currently, owners must complete exhaustive behavioral history forms for veterinarians to evaluate canine behavior problems. In some cases, this process may be expedited by incorporating more concise behavioral survey methods to efficiently characterize the nature and severity of presenting behaviors.

This study aimed to investigate the associations between canine behavior change following clinical intervention and canine demographic characteristics, owner personality and owner-dog attachment. A secondary aim was to examine the usefulness of an existing behavioral assessment questionnaire (the Canine Behavioral Assessment and Research Questionnaire, C-BARQ) as a clinical diagnostic tool and a method of evaluating treatment outcomes.

## Materials and Methods

Dogs referred to the Behavior Medicine Service of the Ryan Veterinary Hospital at the University of Pennsylvania between July 2013 and January 2015 were eligible to participate in the study. Owners were informed of the study while booking an appointment. They were then emailed a consent form, the C-BARQ and a behavior questionnaire from the Penn Vet Behavior Medicine Service which collected canine demographic data, presenting complaint(s), aggression and bite history, training history, indicators of fear or anxiety and information on the home environment and owner(s) (29).

The veterinary consultation involved a physical examination, direct behavior observation of the patient, and behavioral diagnosis. The dog's behavior during the first 30 min of each consultation was video recorded using two or more cameras situated throughout the room (data not included in the present study). Owners were provided with a treatment plan which could include behavioral modification techniques, environmental changes, safety rules and psychopharmacological medications. The owners were then followed-up by telephone approximately 10 days, 3 months, 6 months and 9 months after the consultation. Within 10 days of attending the veterinary consultation, owners were provided with the Lexington Attachment to Pets Scale (LAPS) and the 10-Item Personality Inventory (TIPI). Additionally, at 3- and 6-months post-consultation, the owners were sent an email link to complete the C-BARQ online. All owners provided informed written consent. The study received ethical approval from the Institutional Review Board of the University of Pennsylvania.

### C-BARQ

The C-BARQ is a 100-item questionnaire that has been used extensively as a measure of canine behavior (12, 30–32) and has demonstrated sound reliability and validity (33). The questionnaire requires owners to describe their dog's response to a range of stimuli by indicating the frequency or severity of specific behaviors. Each question can be answered on a five-point scale that ranges from never (zero) to always (four) for frequency-based questions and none (zero) to serious (four) for severity-based questions. The C-BARQ comprises 14 subscales:

- Stranger-directed aggression–Threatening or aggressive responses to strangers approaching or invading the dog's or owner's personal space, territory, or home range.
- Owner-directed aggression–Threatening or aggressive responses to the owner or other members of the household when challenged, manhandled, stared at, stepped over, or when approached while in possession of food or objects.
- Dog-directed aggression–Threatening or aggressive responses when approached directly by unfamiliar dogs.
- Dog rivalry–Aggressive or threatening responses to other familiar dogs in the household.
- Stranger-directed fear–Fearful or wary responses when approached directly by strange or unfamiliar people.
- Nonsocial fear–Fearful or wary responses to sudden or loud noises, traffic, and unfamiliar objects and situations.
- Dog-directed fear–Fearful or wary responses when approached directly by unfamiliar dogs.
- Touch sensitivity–Fearful or wary responses to potentially painful or uncomfortable procedures, including bathing, grooming, nail-clipping, and veterinary examinations.
- Separation-related behavior–Vocalizing and/or destructive behavior when separated from the owner, including autonomic signs of anxiety—restlessness, loss of appetite, trembling, and excessive salivation.
- Attachment and attention-seeking–Maintains close proximity to the owner or other members of the household, solicits affection or attention, becomes agitated when the owner gives attention to third parties.
- Trainability–Willingness to attend to the owner, obey simple commands, fetch objects, respond positively to correction, and ignore distracting stimuli.
- Chasing–Pursues cats, birds, and/or other small animals, given the opportunity.
- Excitability–Reaction to potentially exciting or arousing events, such as going for walks or car trips, doorbells, arrival of visitors, or the owner arriving home; difficulty settling down after such events.
- Energy level–Level of energetic, boisterous, and/or playful behavior.

Subscale scores are calculated as the average of all questions within the subscale with a possible range of 0–4. With the exception of trainability, a higher score is indicative of less desirable behavior. If an owner had not observed their dog in the described scenario, they were instructed to leave the question blank (34). If more than 25% of questions in a subscale were left unanswered, the subscale was coded as missing.

### Lexington Attachment to Pets Scale (LAPS)

The LAPS questionnaire was used to measure the strength of owner-dog attachment. It includes 23 questions which can be answered on four-point response scales. Possible responses range from strongly disagree (zero) to strongly agree (three) and total scores range from zero to 69. Two items in the questionnaire represent weak attachment and were reverse scored (35).

### Ten-Item Personality Inventory (TIPI)

The TIPI is a 10-item questionnaire that assesses human personality based on the “Big Five” personality framework (36). The “Big Five” includes the most basic dimensions of personality: extraversion (e.g., outgoing, enthusiastic), agreeableness (e.g., appreciative, considerate), conscientiousness (responsible, reliable), neuroticism or emotional stability (e.g., anxious, unstable), and openness to experiences (e.g., curious, imaginative) (37). The TIPI includes a list of 10 pairs of personality traits. Dog owners had to indicate the extent to which they believed each characteristic was applicable to them, with possible answers ranging from one (strongly disagree) to seven (agree strongly). The score on each pair of adjectives are averaged to produce a score for the five subscales. The TIPI has demonstrated adequate validity and reliability (36), and has been used previously in similar cohorts (19).

### Statistical Analysis

Data were assessed for normality using visual inspection of the distribution and the Shapiro-Wilk test. Continuous variables that were normally distributed are reported as mean ± standard deviation. The C-BARQ data were not normally distributed so the results are reported as median ± range. Categorical variables are reported using frequency. Spearman's rank coefficients were calculated to determine the correlation between veterinary behavior diagnoses and C-BARQ subscales. Mixed effects models were used to analyze the change in C-BARQ subscale scores following clinical intervention with a sandwich variance structure to account for non-normality. Time was considered a fixed effect and the participant was considered a random effect. To examine the influence of human and canine characteristics on the likelihood of positive treatment outcomes, binary logistic regression models were employed. A reduction in C-BARQ subscale score was coded as a positive treatment outcome for all subscales except trainability where an increased score was considered a positive treatment outcome. Separate models were conducted for each C-BARQ subscale at 3- and 6-months. LAPS score, owner personality scores, baseline C-BARQ score (as an indicator of behavior at baseline), canine age, sex and weight were included as independent variables. The C-BARQ subscale “Dog Rivalry” was excluded due to the small number of cases for this scale (owners of single dogs are not able to answer questions related to “Dog Rivalry”). Statistical analyses were performed in commercially available statistical software (STATA, IBM SPSS Statistics for Windows, version 24). Statistical significance was set at *p* < 0.05.

## Results

### Missing Values

131 dog owners enrolled in the study, provided baseline C-BARQ data, and attended a veterinary behavior consultation. Twelve dog owners did not complete the LAPS and the TIPI. Thirty-three dog owners failed to complete the C-BARQ at 3-months and 44 did not provide C-BARQ data at 6-months.

Individual C-BARQ subscales were coded as missing if owners had not observed their dog in the described situation. At baseline, dog rivalry (49.6%), chasing (16.0%), dog-directed fear (13.0%), and dog-directed aggression (12.2%) had the most missing data. At 3- and 6-months, dog rivalry (3-months = 50.0%, 6-months = 50.6%), dog-directed fear (3-months = 23.5%, 6-months = 17.2%) and dog-directed aggression (3-months = 22.4%, 6-months = 18.4%) had the highest percentage of missing values.

### Demographic Data

The dogs' presenting complaints, behavioral diagnoses and median baseline C-BARQ subscale scores are presented in Table 1. Considering owner-attachment, owners reported a mean LAPS score of 50.5 (SD 9.9, range 25–69). The mean extraversion score was 4.6 (SD 1.6, range 1.0–6.0). Mean agreeableness was 5.6 (SD 1.1, range 1.5–7.0), mean conscientiousness was 6.0 (SD 1.1, range 1.5–7.0), mean emotional stability was 4.9 (SD 1.4, range 1.5–7.0) and the mean score for openness to experiences was 5.5 (SD 1.1, range 2.0–7.0).

**Table 1:** Presenting complaint, veterinarian behavioral diagnoses and median + range of C-BARQ subscale scores at baseline (*n* = 131).

**Category**		**%**	***n***
Presenting	Stranger aggression	38.2	50
complaint	Separation issues	13.0	17
	Dog aggression (unfamiliar)	12.2	16
	Owner aggression	11.5	15
	Interdog aggression (familiar)	9.9	13
	Can't determine/unclear	4.6	6
	Generalized fear	3.8	5
	Stranger-directed fear	2.3	3
	Touch-related aggression	1.5	2
	Excitement	1.5	2
	Attention	0.8	1
Diagnoses ^a^	Displacement behaviors	88.5	116
	Generalized anxiety disorder	80.9	106
	Fear-related aggression toward unfamiliar people	79.4	104
	Fear-related aggression toward unfamiliar dogs	68.7	90
	Resource guarding	55.7	73
	Noise fear	53.4	70
	Territorial aggression	50.4	66
	Confinement anxiety	32.8	43
	Separation anxiety	32.8	43
	Aggression toward familiar people	32.1	42
	Storm fear	28.2	37
	Familiar dog aggression	26.7	35
	Hyperattachment (to owner)	24.4	32
	Inappropriate social skills	19.8	26
	Fearful behavior around people	14.5	19
	Pain-related aggression	7.6	10
	Fearful behavior at the vet	6.9	9
	Other behavioral diagnoses	6.1	8
	Environmental fear	5.3	7
	Predatory aggression	4.6	6
	Redirected aggression	3.8	5
	House soiling	3.8	5
	Excessive licking of surfaces	3.1	4
	Fearful behavior around dogs	2.3	3
	Pica	1.5	2
C-BARQ	Stranger-directed aggression	1.11 (0.00-3.80) ^b^	119
	Owner-directed aggression	0.00 (0.00-3.50) ^b^	127
	Dog-directed aggression	1.50 (0.00-4.00) ^b^	115
	Dog-directed fear	1.00 (0.00-4.00) ^b^	114
	Dog rivalry (familiar dog aggression)	0.50 (0.00-4.00) ^b^	66
	Trainability	2.27 (0.00-3.63) ^b^	126
	Chasing	3.00 (0.00-4.00) ^b^	110
	Stranger-directed fear	0.71 (0.00-4.00) ^b^	124
	Non-social fear	1.00 (0.00-3.67) ^b^	122
	Separation-related behavior	0.75 (0.00-3.67) ^b^	119
	Touch sensitivity	1.00 (0.00-4.00) ^b^	118
	Excitability	2.33 (0.20-4.00) ^b^	129
	Attachment and attention-seeking	2.50 (0.67-4.00) ^b^	126
	Energy	2.00 (0.00-4.00) ^b^	131

*n indicates the number of dogs with each presenting complaint and diagnoses. For C-BARQ data, n shows the number of dogs with valid baseline data for this subscale*.

a *Dogs could have multiple behavioral diagnoses*.

b *Data are presented as median (range)*.

### Veterinary Diagnoses and C-BARQ Subscales

Most of the veterinary behavioral diagnoses were moderately correlated with the corresponding C-BARQ subscale (Table 2). Diagnosis of fear-related aggression toward unfamiliar people was moderately correlated with the C-BARQ scale stranger-directed aggression (*r*_s_ = 0.50, *p* < 0.001). Significant correlations were also found between diagnoses for aggression toward familiar people, unfamiliar dogs and familiar dogs and the C-BARQ subscales owner-directed aggression (*r*_s_ = 0.61, *p* < 0.001), dog-directed aggression (*r*_s_ = 0.48, *p* < 0.001) and dog rivalry (*r*_s_ = 0.49, *p* = 0.001), respectively. Diagnoses of environmental fear was associated with non-social fear (*r*_s_ = 0.40, *p* < 0.001). A moderate correlation was found between veterinary diagnosis of separation anxiety and the C-BARQ subscale for separation-related behavior (*r*_s_ = 0.53, *p* < 0.001).

**Table 2:** Spearman's rank correlation coefficients (*r*_s_) between veterinarian behavioral diagnoses and C-BARQ subscale scores at baseline (*n* = 131).

	**C-BARQ subscales**													
**Diagnoses**	**Stranger- directed aggression**	**Owner- directed aggression**	**Dog- directed aggression**	**Dog rivalry**	**Stranger- directed fear**	**Nonsocial fear**	**Dog- directed fear**	**Touch sensitivity**	**Separation-related behavior**	**Attachment**	**Trainability**	**Chasing**	**Excitability**	**Energy level**
Fear aggression unfamiliar people	0.50 (*p* < 0.001)^a^	—	—	—	0.25 (*p* = 0.03)	—	—	—	—	—	—	—	—	0.25 (*p* = 0.03)
Aggression familiar people	—	0.61 (*p* < 0.01) ^a^	—	—	—	—	—	0.39 (*p* = 0.001)	—	—	—	—	0.24 (p = 0.04)	—
Fear aggression unfamiliar dogs	—	—	0.48 (*p* < 0.001)^a^	—	—	—	—	—	—	—	—	—	—	—
Resource guarding	0.26 (*p* = 0.03)	0.29 (*p* = 0.01)	—	0.56 (*p* < 0.001) ^a^	—	—	—	—	—	—	—	—	—	—
Familiar dog aggression	—	—	—	0.49 (*p* = 0.001) ^a^	—	—	—	—	—	—	—	—	−0.25 (*p* = 0.03)	−0.32 (*p* = 0.004)
Generalized anxiety disorder	—	—	—	—	0.32 (*p* = 0.001)	—	—	—	—	—	—	0.28 (*p* = 0.02)	0.26 (*p* = 0.02)	—
Noise fear	0.25 (*p* = 0.03)	—	—	—	0.34 (*p* = 0.01)	0.27 (0.02)	—	0.25 (*p* = 0.04)	0.29 (*p* = 0.01)	—	—	—	0.35 (*p* = 0.001)	—
Environmental fear	—	—	—	0.34 (*p* = 0.03)	0.27 (*p* = 0,02)	0.40 (*p < * 0.001) ^a^	—	—	—	—	—	—	—	—
Storm fear	—	—	—	—	0.25 (*p* = 0.03)	0.35 (*p* = 0.002)	—	—	—	—	—	—	—	—
Territorial aggression	0.35 (*p* = 0.002)	—	0.24 (*p* = 0.05)	—	—	—	—	—	—	—	—	—	0.25 (*p* = 0.03)	—
Separation anxiety	—	—	—	—	—	—	0.31 (*p* = 0.01)	—	0.53 (*p* < 0.001) ^a^	—	−0.28 (*p* = 0.01)	—	—	—
Confinement anxiety	—	0.23 (*p* = 0.04)	—	—	—	—	0.33 (*p* = 0.01)	—	0.38 (*p* = 0.001)	—	—	—	—	—
Hyperattachment to owner	—	—	—	—	—	—	—	—	—	0.29 (*p* = 0.01)	—	—	—	—
Inappropriate social skills	—	—	—	0.50 (*p* = 0.001) ^a^	—	—	—	—	—	—	—	—	—	—
Displacement behaviors	—	—	—	—	—	—	—	—	−0.29 (*p* = 0.01)	—	—	—	—	—
Redirected aggression	—	—	—	—	—	—	—	—	—	−0.26 (*p* = 0.02)	—	—	—	—
Fearful behavior people	—	—	—	—	0.25 (*p* = 0.04)	0.35 (*p* = 0.002)	—	—	—	—	—	—	−0.23 (*p* = 0.04)	—
Pain-related aggression	—	—	—	—	—	—	—	—	—	—	—	—	—	0.29 (*p* = 0.01)

*Only statistically significant correlations (p < 0.05) are displayed*.

a *Indicates the correlation is of moderate strength (0.4–0.6)*.

Several miscellaneous C-BARQ items were significantly associated with veterinary diagnoses. House soiling was weakly associated with the C-BARQ item “urinates against objects/furnishings in your home” (*r*_s_ = 0.32, *p* = 0.01). Excessive licking of surfaces and pica were weakly associated with “licks people or objects excessively” (*r*_s_ = 0.24, *p* = 0.04, *r*_s_ = 0.25, *p* = 0.03, respectively).

### Effect of Veterinary Intervention on C-BARQ Subscale Scores

Mixed effects models highlighted statistically significant reductions in chasing (coefficient −0.04, *p* = 0.02), separation-related behavior (coefficient −0.04, *p* = 0.01) and energy (coefficient −0.04, *p* = 0.05) (Table 3). Trainability was significantly increased following clinical intervention (coefficient 0.03, *p* = 0.01). There were no significant differences in any of the other C-BARQ subscales.

**Table 3:** Mixed effects models examining the change in C-BARQ subscale scores following clinical behavioral intervention.

**C-BARQ subscale**	**Coefficient (95% CI)**	***P*-value**
Stranger-directed aggression	0.002 (−0.03, 0.03)	*p* = 0.92
Owner-directed aggression	−0.01 (−0.02, 0.01)	*p* =0.47
Dog-directed aggression	0.02 (−0.03, 0.06)	*p* = 0.47
Dog-directed fear	−0.01 (−0.06, 0.04)	*p* = 0.64
Dog rivalry	−0.01 (−0.06, 0.04)	*p* = 0.71
Trainability	0.03 (0.01, 0.05)	*p* = 0.01^*^
Chasing	−0.04 (−0.07, −0.01)	*p* = 0.02^*^
Stranger-directed fear	0.01 (−0.03, 0.05)	*p* = 0.57
Separation-related behavior	−0.04 (−0.07, −0.01)	*p* = 0.01^*^
Touch sensitivity	−0.01 (−0.04, 0.02)	*p* = 0.56
Excitability	−0.004 (−0.03, 0.02)	*p* = 0.74
Attachment and attention-seeking	−0.02 (−0.05, 0.003)	*p* = 0.09
Energy level	−0.04 (−0.07, −0.0004)	*p* = 0.05^*^
Non-social fear	−0.01 (−0.03, 0.01)	*p* = 0.41

* *Denotes statistical significance (p < 0.05)*.

### Influence of Owner and Canine Characteristics on C-BARQ Subscale Scores

The associations between owner and canine characteristics and behavior change at 3- and 6-months are shown in Tables 4, 5, respectively.

**Table 4:** Logistic regression describing the associations between positive treatment outcomes at 3-months and owner and canine characteristics.

**C-BARQ subscale**	**Cases/*n*^a^**	**Age ^b^**	**Sex ^c^**	**Weight ^d^**	**Baseline C-BARQ score**	**Owner attachment**	**Owner personality**				
							**Extraversion**	**Agreeableness**	**Conscientiousness**	**Emotional stability**	**Openness to experience**
Stranger-directed aggression	38/76	0.27 (0.09–0.86) *p* = 0.03^*^	0.61 (0.18–2.15) *p =* 0.45	3.30 (1.14–9.61) *p =* 0.03^*^	2.94 (1.40–6.14) *p =* 0.04^*^	1.02 (0.95–1.09) *p =* 0.68	1.07 (0.71–1.60) *p =* 0.74	0.69 (0.40–1.18) *p =* 0.17	0.47 (0.24–0.94) *p =* 0.03^*^	1.83 (0.98–3.41) *p =* 0.06	1.50 (0.73–3.09) *p =* 0.27
Owner-directed aggression	22/89	1.15 (0.37–3.59) *p =* 0.80	0.62 (0.15–2.54) *p =* 0.51	0.71 (0.21–2.42) *p =* 0.59	6.48 (1.97–21.32) *p =* 0.002^*^	0.97 (0.90–1.03) *p =* 0.30	0.86 (0.58–1.28) *p =* 0.46	0.80 (0.46–1.39) *p =* 0.43	0.66 (0.36–1.24) *p =* 0.20	1.47 (0.82–2.61) *p =* 0.19	0.86 (0.49–1.51) *p =* 0.60
Dog-directed aggression	30/70	0.60 (0.21–1.72) *p =* 0.34	1.57 (0.51–4.88) *p =* 0.44	2.00 (0.72–5.54) *p =* 0.18	1.64 (0.97–2.78) *p =* 0.07	1.05 (0.98–1.11) *p =* 0.16	1.10 (0.79–1.54) *p =* 0.57	0.75 (0.45–1.24) *p =* 0.26	0.98 (0.62–1.56) *p =* 0.94	1.03 (0.69–1.54) *p =* 0.90	0.85 (0.50–1.45) *p =* 0.55
Stranger-directed fear	21/80	0.89 (0.27–3.00) *p =* 0.85	1.33 (0.32–5.48) *p =* 0.70	1.31 (0.39–4.42) *p =* 0.66	3.78 (1.84–7.77) *p < * 0.01	1.04 (0.96–1.13) *p =* 0.33	1.44 (0.90–2.30) *p =* 0.13	0.76 (0.43–1.35) *p =* 0.35	1.33 (0.71–2.48) *p =* 0.37	0.80 (0.43–1.49) *p =* 0.80	1.10 (0.51–2.34) *p =* 0.81
Nonsocial fear	33/80	0.25 (0.07–0.92) *p =* 0.04^*^	0.88 (0.24–3.15) *p =* 0.84	3.28 (1.01–10.62) *p =* 0.05^*^	7.35 (2.65–20.38) *p < * 0.01^*^	0.97 (0.91–1.04) *p =* 0.43	1.84 (1.15–2.95) *p =* 0.01^*^	0.95 (0.53–1.70) *p =* 0.86	0.89 (0.52–1.52) *p =* 0.66	1.11 (0.67–1.86) *p =* 0.69	0.99 (0.50–1.95) *p =* 0.97
Dog-directed fear	23/70	0.44 (0.12–1.69) *p =* 0.23	1.92 (0.48–7.65) *p =* 0.36	2.31 (0.71–7.50) *p =* 0.16	2.18 (1.17–4.04) *p =* 0.01^*^	1.03 (0.95–1.10) *p =* 0.52	1.49 (0.98–2.26) *p =* 0.06	0.81 (0.47–1.41) *p =* 0.46	0.70 (0.39–1.24) *p =* 0.22	1.75 (0.97–3.18) *p =* 0.07	0.48 (0.25–0.95) *p =* 0.03^*^
Touch sensitivity	32/80	0.57 (0.20–1.56) *p =* 0.27	1.27 (0.39–4.13) *p =* 0.70	0.42 (0.15–1.17) *p =* 0.10	1.92 (1.04–3.51) *p =* 0.04^*^	1.08 (1.00–1.16) *p =* 0.04^*^	1.74 (1.15–2.65) *p =* 0.01^*^	0.85 (0.52–1.41) *p =* 0.53	0.95 (0.57–1.59) *p =* 0.85	1.38 (0.83–2.29) *p =* 0.22	0.90 (0.51–1.59) *p =* 0.72
Separation-related behavior	45/87	1.06 (0.43–2.62) *p =* 0.90	1.89 (0.66–5.41) *p =* 0.24	1.00 (0.41–2.40) *p =* 0.99	3.10 (1.60–5.98) *p =* 0.01^*^	1.07 (1.01–1.13) *p =* 0.03^*^	0.93 (0.68–1.29) *p =* 0.68	1.01 (0.65–1.57) *p =* 0.97	0.93 (0.58–1.49) *p =* 0.75	1.01 (0.67–1.52) *p =* 0.97	1.21 (0.73–2.00) *p =* 0.46
Attachment and attention-seeking	43/88	0.32 (0.13–0.78) *p =* 0.01^*^	0.32 (0.11–0.92) *p =* 0.03^*^	1.76 (0.75–4.15) *p =* 0.20	2.36 (1.17–4.77) *p =* 0.02^*^	0.98 (0.93–1.03) *p =* 0.46	0.93 (0.67–1.28) *p =* 0.64	1.00 (0.64–1.57) *p =* 1.00	1.47 (0.91–2.39) *p =* 0.12	1.01 (0.67–1.52) *p =* 0.95	1.06 (0.61–1.86) *p =* 0.83
Trainability	61/90	0.57 (0.23–1.42) *p =* 0.23	0.62 (0.21–1.84) *p =* 0.39	1.98 (0.80–4.92) *p =* 0.14	0.20 (0.07–0.57) *p < * 0.01^*^	1.00 (0.95–1.06) *p =* 0.89	0.75 (0.53–1.07) *p =* 0.11	0.66 (0.39–1.12) *p =* 0.13	1.11 (0.70–1.77) *p =* 0.66	1.12 (0.74–1.71) *p =* 0.60	1.09 (0.62–1.91) *p =* 0.77
Chasing	40/79	0.62 (0.24–1.63) *p =* 0.34	2.03 (0.68–6.04) *p =* 0.20	1.67 (0.66–4.23) *p =* 0.23	1.88 (1.08–3.29) *p =* 0.03^*^	1.04 (0.99–1.10) *p =* 0.15	0.82 (0.58–1.14) *p =* 0.23	0.97 (0.60–1.58) *p =* 0.91	0.91 (0.57–1.44) *p =* 0.69	1.24 (0.82–1.86) *p =* 0.31	1.08 (0.66–1.77) *p =* 0.75
Excitability	40/91	0.31 (0.11–0.86) *p =* 0.03^*^	3.20 (1.09–9.33) *p =* 0.03^*^	2.17 (0.85–5.56) *p =* 0.11	3.75 (1.71–8.22) *p < * 0.01^*^	0.99 (0.93–1.04) *p =* 0.63	0.97 (0.70–1.34) *p =* 0.84	1.01 (0.63–1.63) *p =* 0.97	0.82 (0.52–1.28) *p =* 0.38	0.89 (0.59–1.32) *p =* 0.55	1.14 (0.69–1.88) *p =* 0.60
Energy level	38/94	1.39 (0.56–3.47) *p =* 0.48	0.72 (0.25–2.04) *p =* 0.53	2.09 (0.89–4.95) *p =* 0.09	2.64 (1.46–4.77) *p < * 0.01^*^	1.01 (0.96–1.07) *p =* 0.73	1.06 (0.76–1.47) *p =* 0.74	1.54 (0.94–2.53) *p =* 0.09^*^	1.32 (0.81–2.14) *p =* 0.26	0.79 (0.53–1.20) *p =* 0.27	0.77 (0.46–1.27) *p =* 0.30

Data are shown as odds ratio (95% confidence intervals)

a *Cases indicates the number of dogs who showed a decrease in the C-BARQ subscale between baseline and 3-months for all subscales except trainability where cases reflect the number of dogs that showed an increase in the subscale between baseline and 3-months*.

b *Dogs that were ≤ the median age (36 months) were considered as the referent category*.

c *Female dogs were the referent category*.

d *Dogs that weighed ≤ median weight (23.2 kgs) were considered as the referent category*.

* *Denotes statistical significance (p < 0.05)*.

**Table 5:** Logistic regression describing the associations between positive treatment outcomes at 6-months and owner and canine characteristics.

**C-BARQ subscale**	**Cases/*n*^a^**	**Age ^b^**	**Sex ^c^**	**Weight ^d^**	**Baseline C-BARQ**	**Owner attachment**	**Owner personality**				
							**Extraversion**	**Agreeableness**	**Conscientiousness**	**Emotional stability**	**Openness to experience**
Stranger-directed aggression	29/65	1.22 (0.36–4.07) *p =* 0.75	0.38 (0.10–1.48) *p =* 0.16	1.13 (0.39–3.23) *p =* 0.82	1.67 (0.85–3.28) *p =* 0.14	1.11 (1.03–1.20) *p =* 0.01^*^	1.10 (0.73–1.66) *p =* 0.66	0.92 (0.54–1.58) *p =* 0.76	0.45 (0.22–0.94) *p =* 0.03^*^	1.04 (0.65–1.66) *p =* 0.89	1.02 (0.50–2.09) *p =* 0.96
Owner-directed aggression	22/76	1.32 (0.33–5.33) *p =* 0.70	2.13 (0.44–10.24) *p =* 0.35	0.25 (0.06–1.10) *p =* 0.07	6.76 (1.57–29.23) *p =* 0.01^*^	0.94 (0.88–1.02) *p =* 0.13	0.76 (0.50–1.17) *p =* 0.21	1.46 (0.72–2.93) *p =* 0.29	0.90 (0.49–1.65) *p =* 0.73	1.28 (0.67–2.45) *p =* 0.45	0.70 (0.36–1.35) *p =* 0.28
Dog-directed aggression	25/63	2.45 (0.74–8.06) *p =* 0.14	1.98 (0.52–7.60) *p =* 0.32	0.51 (0.16–1.66) *p =* 0.27	1.38 (0.79–2.41) *p =* 0.26	0.94 (0.87–1.00) *p =* 0.06	1.25 (0.87–1.79) *p =* 0.23	0.99 (0.54–1.83) *p =* 0.99	0.67 (0.39–1.16) *p =* 0.15	1.22 (0.75–2.00) *p =* 0.42	0.89 (0.47–1.70) *p =* 0.73
Stranger-directed fear	26/68	1.21 (0.65–2.27) *p =* 0.81	0.66 (0.18–2.43) *p =* 0.53	1.01 (0.33–3.05) *p =* 0.58	2.81 (1.54–5.12) *p =* 0.01^*^	1.03 (0.95–1.10) *p =* 0.51	1.07 (0.74–1.55) *p =* 0.72	0.59 (0.33–1.04) *p =* 0.07	0.98 (0.58–1.63) *p =* 0.93	1.06 (0.65–1.73) *p =* 0.81	1.21 (0.65–2.27) *p =* 0.55
Nonsocial fear	27/70	0.53 (0.11–2.63) *p =* 0.44	0.38 (0.08–1.79) *p =* 0.22	3.78 (0.92–15.64) *p =* 0.07	6.58 (2.35–18.39) *p < * 0.01^*^	0.96 (0.89–1.04) *p =* 0.28	1.60 (0.97–2.62) *p =* 0.07	0.53 (0.26–1.06) *p =* 0.07	1.95 (0.89–4.27) *p =* 0.10	1.53 (0.86–2.73) *p =* 0.15	1.09 (0.49–2.44) *p =* 0.83
Dog-directed fear	21/62	1.69 (0.40–7.23) *p =* 0.48	1.59 (0.34–7.40) *p =* 0.56	1.57 (0.36–6.81) *p =* 0.55	2.80 (1.42–5.50) *p =* 0.003^*^	0.95 (0.87–1.03) *p =* 0.18	0.73 (0.48–1.13) *p =* 0.16	1.21 (0.61–2.43) *p =* 0.59	0.55 (0.30–1.02) *p =* 0.06	1.13 (0.65–1.95) *p =* 0.67	0.78 (0.40–1.53) *p =* 0.46
Touch sensitivity	28/65	0.81 (0.25–2.59) *p =* 0.72	1.35 (0.37–4.91) *p =* 0.65	1.19 (0.41–3.47) *p =* 0.76	2.11 (1.10–4.03) *p =* 0.03^*^	1.05 (0.98–1.12) *p =* 0.15	1.47 (0.97–2.23) *p =* 0.07	0.92 (0.54–1.59) *p =* 0.77	1.16 (0.67–1.99) *p =* 0.61	1.55 (0.89–2.70) *p =* 0.12	0.73 (0.38–1.39) *p =* 0.34
Separation-related behavior	30/70	1.21 (0.41–3.61) *p =* 0.73	0.36 (0.11–1.17) *p =* 0.09	1.22 (0.44–3.40) *p =* 0.71	0.43 (0.22–0.83) *p =* 0.01^*^	0.98 (0.92–1.04) *p =* 0.45	1.13 (0.80–1.62) *p =* 0.49	0.83 (0.51–1.33) *p =* 0.43	1.07 (0.65–1.75) *p =* 0.79	1.10 (0.70–1.72) *p =* 0.69	1.18 (0.68–2.07) *p =* 0.56
Attachment and attention-seeking	42/76	0.26 (0.08–0.86) *p =* 0.03^*^	1.20 (0.38–3.75) *p =* 0.75	3.07 (0.96–9.78) *p =* 0.06	3.49 (1.45–8.40) *p =* 0.01^*^	1.05 (0.99–1.12) *p =* 0.09	1.29 (0.91–1.84) *p =* 0.16	0.68 (0.37–1.22) *p =* 0.19	1.03 (0.64–1.67) *p =* 0.90	0.84 (0.54–1.30) *p =* 0.42	1.10 (0.62–1.93) *p =* 0.75
Trainability	51/77	0.25 (0.07–0.89) *p =* 0.03^*^	1.55 (0.45–5.30) *p =* 0.48	1.13 (0.34–3.71) *p =* 0.85	0.40 (0.13–1.20) *p =* 0.10	1.10 (1.02–1.18) *p =* 0.02^*^	0.87 (0.60–1.26) *p =* 0.45	0.91 (0.54–1.56) *p =* 0.74	1.16 (0.69–1.93) *p =* 0.58	0.91 (0.58–1.43) *p =* 0.68	1.33 (0.73–2.43) *p =* 0.35
Chasing	34/63	0.98 (0.23–4.10) *p =* 0.98	0.66 (0.19–2.37) *p =* 0.53	0.73 (0.22–2.42) *p =* 0.60	2.39 (1.22–4.66) *p =* 0.01^*^	0.98 (0.92–1.05) *p =* 0.57	0.89 (0.62–1.28) *p =* 0.52	0.61 (0.32–1.15) *p =* 0.12	0.61 (0.36–1.02) *p =* 0.06	1.50 (0.92–2.43) *p =* 0.10	0.93 (0.49–1.76) *p =* 0.83
Excitability	35/76	0.48 (0.16–1.46) *p =* 0.20	4.38 (1.28–15.06) *p =* 0.02^*^	2.53 (0.87–7.38) *p =* 0.09	2.34 (1.09–5.03) *p =* 0.03^*^	1.02 (0.96–1.09) *p =* 0.46	0.77 (0.55–1.09) *p =* 0.14	1.25 (0.76–2.07) *p =* 0.38	0.87 (0.53–1.43) *p =* 0.59	0.83 (0.53–1.23) *p =* 0.39	1.29 (0.75–2.22) *p =* 0.36
Energy level	31/79	1.33 (0.43–4.06) *p =* 0.62	2.20 (0.70–6.95) *p =* 0.18	1.19 (0.45–3.19) *p =* 0.73	2.32 (1.20–4.48) *p =* 0.01^*^	0.96 (0.91–1.02) *p =* 0.20	1.02 (0.73–1.42) *p =* 0.91	1.28 (0.76–2.16) *p =* 0.36	1.08 (0.65–1.80) *p =* 0.77	0.77 (0.50–1.19) *p =* 0.24	0.76 (0.45–1.29) *p =* 0.31

Data are shown as odds ratio (95% confidence intervals)

a *Cases indicates the number of dogs who showed a decrease in the C-BARQ subscale between baseline and 6-months for all subscales except trainability where cases reflect the number of dogs that showed an increase in the subscale between baseline and 6-months*.

b *Dogs that were ≤ the median age (36 months) were considered as the referent category*.

c *Female dogs were the referent category*.

d *Dogs that weighed ≤ median weight (23.2 kgs) were considered as the referent category*.

* *Denotes statistical significance (p < 0.05)*.

#### Canine Behavior at Baseline

Behavior at baseline was consistently associated with treatment outcomes at 3-months with significant associations for all subscales except dog-directed aggression. With the exception of trainability, a one-point increase in baseline C-BARQ subscale scores were associated with greater odds of a decrease within 3-months, i.e., dogs with less desirable behavior at baseline were more likely to display a decrease in the behavior at 3-months. The largest associations were seen for non-social fear and owner-directed aggression, where a one-point increase at baseline was associated with 7.35 (95% CI 2.65–20.38) and 6.48 (95% 1.97–21.32 CI) times the odds of decreased behavior at 3 months, respectively. Considering trainability, a one-point increase at baseline was associated with 80% lower odds of increased trainability at 3-months (OR 0.20, 95% CI 0.07–0.50), i.e., dogs that were more trainable at baseline were less likely to exhibit increased trainability at 3-months.

At 6-months, baseline behavior was significantly associated with all C-BARQ subscales except stranger-directed aggression, dog-directed aggression and trainability. Again, the largest odds ratios were found for non-social fear (OR 6.58, 95% CI 2.35–18.39) and owner-directed aggression (OR 6.76, 95% CI 1.57–29.23).

The association between separation-related behavior at baseline and decreased behavior at 6-months differed from the association observed at 3-months. A higher score at baseline (i.e., less desirable behavior) was associated with 3.10 times the odds of decreased separation-related behavior (95% CI 1.60–5.98) at 3-months, whereas at 6-months the opposite was true (OR 0.43, 95% CI 0.22–0.83).

#### Canine Age, Sex, and Weight

Age was significantly associated with behavior change for attention-seeking behavior, stranger-directed aggression, non-social fear, excitability and trainability. Considering attention-seeking behavior, dogs aged over the median age (36 months) at baseline had reduced odds of decreased attention-seeking behavior at both 3 and 6-months post-consultation (OR 0.32, 95% CI 0.13–0.78 and OR 0.26, 95% CI 0.08–0.86, respectively). At 3-month measurements only, dogs over 36 months also had significantly lower odds of decreased stranger-directed aggression (OR 0.27, 95% CI 0.09–0.86), non-social fear (OR 0.25, 95% CI 0.07–0.92) and excitability (OR 0.31, 95% CI 0.11–0.86). At 6-months only, trainability was significantly associated with age in that dogs aged above the median age had a 75% reduction in the relative odds of exhibiting increased trainability (OR 0.25, 95% CI 0.07–0.89).

Canine sex was significantly associated with excitability and attention-seeking behavior. Female dogs had 3.20 times the odds of decreased excitability within 3-months (OR 3.20, 95% CI 1.09–9.22) and 4.38 times the odds within 6-months (OR 4.38, 95% CI 1.28–15.06). Females were also 68% less likely than males to exhibit decreased attention-seeking behavior within 3-months (OR 0.32, 95% 0.11–0.92).

Weight was associated with behavior change for stranger-directed aggression and non-social fear at 3-months only. For every one-kilogram increase, dogs had 3.30 times the odds of decreased stranger-directed aggression (95% CI 1.14–9.61) and 3.28 times the odds of decreased non-social fear (95% CI 1.01–10.62). There were no significant associations between weight and behavior change at 6-month measurements.

#### Owner Personality

Several of the Big Five personality traits were associated with behavior change for some C-BARQ behavioral categories. Owner conscientiousness was negatively associated with change in stranger-directed aggression at 3- and 6-months, with a one-point increase in conscientiousness corresponding to 53% lower odds of decreased stranger-directed aggression at 3-months (OR 0.47, 95% CI 0.24–0.94) and 55% lower odds at 6-months (OR 0.45, 95% CI 0.22–0.94). Change in dog-directed fear was associated with owners' openness to experience, with every one-point increase bringing a 52% reduction in the odds of decreased dog-directed fear at 3-months (OR 0.48, 95% CI 0.25–0.95). Owner extraversion was positively associated with change in non-social fear and touch sensitivity. For every one-point increase in extraversion, dogs had 84% greater odds of dogs exhibiting decreased non-social fear (OR 1.84, 95% CI 1.15–2.95) and 74% greater odds of decreased touch sensitivity (OR 1.74, 1.15–2.65) at 3-month measurements.

#### Owner Attachment

We found statistically significant but weak associations between the strength of owner attachment and touch sensitivity (OR 1.08, 95% CI 1.00–1.16) and separation-related behavior (OR 1.07, 95% CI 1.01–1.13) at 3-months. Stranger-directed aggression (OR 1.11, 95% CI 1.03–1.20) and trainability (OR 1.10, 95% CI 1.02–1.18) were also associated with owner attachment at 6-months.

## Discussion

In this study, we investigated behavior change following veterinary intervention in canine behavioral medicine cases using an established assessment tool, the C-BARQ (33). Following veterinary consultation, we found a significant increase in trainability and a significant reduction in chasing, separation-related behavior and energy level. As pathological behavioral disorders often cannot be cured due to their complex underlying neurochemical and genetic basis, management through behavior modification is essential (24). To implement behavior modification techniques, dogs must be attentive, responsive to commands and able to ignore distracting stimuli. As such, trainability is vital for the success of clinical intervention and comprises a key feature of many veterinary behavioral treatment plans. The increase in trainability noted in the present study suggests owners could implement behavioral modification techniques and better manage their dog's behavioral problems. The reduction in chasing, separation-related behavior and energy may also be attributed to the increase in trainability. Previous research has found an inverse relationship between trainability and separation-related (38, 39) and boisterous behavior (40). Psychopharmacological medications may also have contributed to the reduction in undesirable behavior, particularly for separation-related behavior as long-term medication is often required (41, 42). Data regarding the use of behavioral medication was not available in the present study.

Behavior change following veterinary intervention was associated with canine demographic characteristics, owner personality and owner-dog attachment. Canine behavior at baseline was the most consistent predictor of treatment outcomes with less desirable baseline behavior associated with an increased likelihood of decreased problem behavior for most behavioral categories. The treatment of behavioral problems is usually thought to become more difficult as the behaviors become more established (23), although it is also feasible that dogs with less desirable behavior at baseline had greater scope for improvement. Veterinary treatment may have better targeted these dogs, thereby more effectively addressing their problem behaviors than dogs with mild behavioral problems. Considering separation-related behavior, the influence of baseline behavior on treatment outcomes varied between 3- and 6-months. Higher (worse) scores for separation-related behavior at baseline were associated with an increased likelihood of decreased behavior at 3-months but a reduced likelihood of decreased behavior at 6-months. Separation-related behavior is a complex condition for which treatment is demanding and progress is usually slow (43). Severe separation anxiety tends to rely on psychopharmacological treatment, although it is rarely curative (41). The effectiveness of medication is likely to be somewhat dependent on its sedative effect (44, 45). Separation anxiety can also have profound effects on the human-dog relationship (38, 41) and owner compliance is a challenge in the treatment of separation-related behavior (46). Fatigue in the provision of treatment may also have contributed to the variation in separation-related behavior between 3- and 6-month treatment outcomes.

Canine age was negatively associated with treatment outcomes for attention-seeking behavior, stranger-directed aggression, non-social fear and excitability at 3-months, and attention-seeking behavior and trainability at 6-months. The inverse relationship between age and the odds of behavioral improvement is logical as trainability and sociability tend to decrease with age (21), problem behaviors are likely to be more established in older dogs, and some problem behaviors are known to increase with age (47, 48).

We also found an association between canine sex and excitability and attention-seeking behavior. Females were more likely to show decreased excitability at 3- and 6-months, and less likely to show decreased attention-seeking behavior at 3-months than male dogs. The difference in treatment outcomes between male and female dogs may reflect underlying personality differences between the sexes. Several studies have shown that male dogs are more bold than female dogs, characterized by an increased willingness to play and lower frequency and intensity of fear (21, 49). A greater prevalence of aggression has also been documented among male dogs while female dogs are more frequently categorized as anxious (13, 22). However, inconsistencies in the literature regarding canine sex and personality indicate that further research is needed (50).

Greater bodyweight (or body size) was associated with decreased stranger-directed aggression and non-social fear at 3-months, although the confidence intervals were relatively large so the results should be interpreted with caution. As the safety risk is inherently greater with increasing bodyweight, it is possible that large dog owners followed the safety rules and veterinary treatment plan more diligently than owners of small dogs, thereby avoiding situations that could trigger undesirable behavior. Strangers are also likely to be more fearful of large dogs exhibiting aggressive or fearful behaviors than small dogs and may have kept a greater distance from them.

Owner personality influenced the likelihood of decreased stranger-directed aggression, non-social fear, touch sensitivity and dog-directed fear. Dogs of owners who scored high on conscientiousness were significantly less likely to exhibit decreased stranger-directed aggression at both 3- and 6-month measurement points. Owner conscientiousness may influence how an owner perceives their dog's behavior and the severity of the behavioral problem. For example, owners who scored high on conscientiousness have been found to rate their dogs as more excitable, less fearful and less aggressive. However, these results may not be generalizable to the current study as the study population did not specifically include dogs with behavioral problems (51). Conscientiousness among dog owners may also be associated with ownership behaviors or lifestyles factors that promote territorial aggression toward unfamiliar people in dogs. Previous research has found no associations between the severity of stranger-directed aggression and owner characteristics, such as previous ownership history, so further research is needed (52). Owner extraversion was positively associated with the likelihood of decreased non-social fear and touch sensitivity within 3-months of veterinary intervention. Individuals with high levels of extraversion tend to be enthusiastic, responsive to social stimuli and value a high volume of social interactions, whereas introverted individuals tend to be reserved, less likely to seek social stimulation and value intimacy over quantity in social relationships (36, 53, 54). In the human-dog relationship, it is possible that introverted owners seek greater companionship from their dogs and may be more resistance to detaching themselves from the dog. However, the relationship between introversion and the strength of human-dog attachment has not received much scientific attention and recent research indicates that extraverted owners may report higher levels of owner-dog attachment (55). Finally, openness to experience among owners was associated with lower odds of decreased dog-directed fear within 3-months. It is possible that owners with higher levels of openness utilized novel training ideologies, such as positive reinforcement, prior to attending the behavior service. Dog owners who scored lower in openness may have relied on historic training methods based on forceful methods and positive punishment, which have been associated with increased fear (56–58). This hypothetical difference in training methods may have affected the potential for behavioral change following veterinary intervention. In accordance, research has shown working dog handlers with low levels of openness were less likely to use novel training tools, such as clickers, compared with handlers who reported higher levels of openness (59). The associations between canine behavior and owner extraversion and openness did not persist at 6-months, so further research is needed to understand the long-term influence of owner personality on behavioral treatment outcomes.

In terms of owner-dog attachment, owners with higher levels of attachment to their dogs were more likely to report decreased touch sensitivity and separation-related behavior within 3-months, and decreased stranger-directed aggression and increased trainability within 6 months. Owners with higher levels of attachment to their dogs may invest more time and energy into managing and treating their dog's behavioral problem. However, the associations between attachment and treatment outcomes were relatively weak and inconsistent between 3- and 6-month measurements so further research is needed.

A secondary aim of the study was to establish the usefulness of the C-BARQ, an existing behavioral assessment tool, for use in veterinary behavioral medicine. We found moderate correlations between most veterinary behavioral diagnoses and the corresponding C-BARQ subscales, supporting the use of C-BARQ as an aid to current diagnostic tools. C-BARQ also showed promise as a method of evaluating behavioral treatment outcomes following veterinary consultation highlighting a statistically significant difference in chasing, separation-related behavior, energy, and trainability. The changes in each subscale were relatively small and most did not reach statistical significance, so it is possible the C-BARQ may not have been sensitive enough to quantify some behavioral changes. The absence of statistically significant differences in most subscales could also reflect a true absence of behavioral change for several reasons: (1) the C-BARQ measures a range of behavioral categories meaning that some behaviors covered by the tool may not have been targeted by the veterinary treatment or perceived as a problem by the owner; (2) many behavioral pathologies cannot be cured due to an underlying genetic component and instead are managed to prevent severe incidents (24); and (3) owners are almost solely responsible for providing treatment in behavioral cases, meaning treatment outcomes are heavily dependent on owner compliance (25, 60, 61). Veterinarians report a lack of compliance, particularly with behavior modification and management techniques, as one of the biggest challenges they face (28).

This study is subject to several limitations. A lack of information was available regarding the final disposition of dogs who did not complete the study meaning that there may have been differential dropout rates based on the dog's response to clinical treatment. It is possible that dogs whose behavior improved rapidly did not complete the study as their owners did not feel the need for ongoing veterinary support. On the other hand, dogs who did not respond to clinical intervention may have dropped out due to euthanasia or rehoming during the study period. This could yield systematic differences between dogs who dropped out and those who completed the study and may have biased results. It is also possible that owner characteristics, such as personality or attachment to the dog, may have influenced the owner's decision not to complete the study. Additional variables, such as the dog's socialization and training history (62–64), may influence behavioral treatment outcomes, although a lack of data precluded us from including these variables in the analysis. The study also lacked information about the owner's perception of their dog's behavior. While dogs with poorer baseline behavior were more likely to show improvement following veterinary treatment, it is possible that the dog's problem behaviors were still at a level that the owner deemed unsatisfactory. Another limitation of the study is the reliance on owner-reported assessments of the dog's behavioral response to veterinary treatment as the ability of dog owners to accurately identify dog behavior is poor (65, 66). However, direct behavioral assessments are subject to their own shortcomings and the C-BARQ has been designed to reduce bias by asking about the dog's response to specific situations using frequency and severity scales (33). The study is also compromised by the relatively small sample size and the missing values on some C-BARQ subscales, particularly dog rivalry, unfamiliar dog aggression and fear. The missing values are presumed to be the result of owners who had not observed their dog in the situation described, but the missing values may also reflect respondent fatigue (67). Finally, the association between human and canine variables, and some behavioral treatment outcomes differed between 3- and 6-months. This may reflect true differences in the canine response to veterinary treatment between the two time points, although the instability in the results mean further research is needed to confirm our findings.

In this sample of dogs who attended a veterinary behavioral service, clinical intervention produced reductions in chasing, separation-related behavior and energy, and increased trainability. Canine behavior at baseline was the most consistent predictor of treatment outcomes with less desirable behavior at baseline associated with greater odds of improvement. Treatment outcomes were also associated with canine age and characteristics of owner personality, namely; conscientiousness, extraversion and openness. This study also provides evidence to support the use of C-BARQ in canine behavioral medical cases.

## Data Availability Statement

The raw data supporting the conclusions of this article will be made available by the authors, without undue reservation.

## Ethics Statement

The studies involving human participants were reviewed and approved by Institutional Review Board, University of Pennsylvania. The patients/participants provided their written informed consent to participate in this study. The animal study was reviewed and approved by Institutional Review Board, University of Pennsylvania. Written informed consent was obtained from the owners for the participation of their animals in this study.

## Author Contributions

CS and JS conceived and designed the study. CS collected the data. LP organized the database and drafted the manuscript. DS and LP conducted the statistical analyses. All authors contributed to manuscript revision, read and approved the submitted version.

## Conflict of Interest

The authors declare that the research was conducted in the absence of any commercial or financial relationships that could be construed as a potential conflict of interest.
